# Simultaneous Detection of Four *Madurella* Species Using Loop-Mediated Isothermal Amplification (LAMP) for Eumycetoma Diagnosis

**DOI:** 10.1007/s11046-025-01019-4

**Published:** 2025-10-29

**Authors:** Isato Yoshioka, Ahmed Hassan Fahal, Doudou Sow, Satoshi Kaneko, Yugo Mori, Sayaka Ban, Takashi Yaguchi

**Affiliations:** 1 Medical Mycology Research Center, Chiba University, Chiba, Japan; 2 Mycetoma Research Centre, University of Khartoum, Khartoum, Sudan; 3 Service de Parasitologie-Mycologie, UFR Sciences de La Santé, Université Gaston Berger, Saint-Louis, Senegal; 4 School of Tropical Medicine and Global Health, Nagasaki University, Nagasaki, Japan; 5 Department of Ecoepidemiology, Institute of Tropical Medicine (NEKKEN), Nagasaki University, Nagasaki, Japan

**Keywords:** Eumycetoma, *Madurella mycetomatis*, Diagnosis, Loop-mediated isothermal amplification, Genomic sequencing, Comparative genome analysis

## Abstract

Eumycetoma is a neglected tropical disease caused primarily by a *Madurella mycetomatis* infection, besides other related species. In this study, we designed a novel loop-mediated isothermal amplification (LAMP) primer set capable of simultaneously detecting four *Madurella* species (*M. mycetomatis*, *M. pseudomycetomatis*, *M. tropicana,* and *M. fahalii*). Genomic sequencing of *M. pseudomycetomatis* strain and comparative genome analysis revealed the candidate genes that were common among and specific to *Madurella* species. The 3 LAMP primer sets targeting these genes detected up to 1 pg of the genomic DNA of all 4 *Madurella* species, exhibiting no cross-reactivity toward other pathogenic fungi. Among these, one primer set showing better reactivity was selected as a candidate used for diagnosis. Therefore, we developed novel primer sets which enabled the simultaneous detection of four *Madurella* species. Our present findings will lead to a faster and simpler diagnostic tool for eumycetoma detection, especially in rural clinical settings.

**Supplementary Information:**

The online version contains supplementary material available at 10.1007/s11046-025-01019-4.

## Short Communication

Mycetoma is a neglected tropical disease caused by microbial infection, resulting in chronic granulomatous inflammation of the subcutaneous tissue discharging grains [1, 2]. Although a wide range of microorganisms, including actinomycetes and fungi, have been reported as causative agents for this disease, the most frequently cited microbe in fungal mycetoma (eumycetoma) is *Madurella mycetomatis* [3, 4]. In addition, all of the known species belonging to the genus *Madurella* (*M. mycetomatis*, *M. pseudomycetomatis*, *M. tropicana* and *M. fahalii*) are known to cause eumycetoma [4]. Due to the presence of small and painless subcutaneous swellings as the initial symptoms of the slowly progressing mycetoma, misdiagnosis sometimes occurs and leads to high morbidity and difficulty in achieving a cure [5]. Therefore, a sensitive detection tool specific to *Madurella* is urgently needed for the early diagnosis of mycetoma, particularly in remote rural settings.

Considering this diagnostic scenario, various molecular methodologies have been developed over the years to detect the causative agent of mycetoma. For example, a polymerase chain reaction (PCR) assay specific to *M. mycetomatis* or *Madurella* species has been published [6, 7]. However, isothermal DNA amplification appears more feasible compared with PCR because an isothermal reaction does not require expensive instrumentation that would otherwise be needed for thermal cycling and/or fluorescence monitoring, both of which may not be available at a low-resource rural hospital [8]. Based on this rationale, we have previously constructed a loop-mediated isothermal amplification (LAMP) system targeting ribosomal DNA in *Madurella* species [9]. Although we have previously described the simple identification of *M. mycetomatis* and *M. fahalii* by a combination of three primer sets (three reactions) [9], the development of a simpler and more versatile system will contribute to the initial screening for *Madurella* by reducing diagnostic costs and enhancing throughput.

In this study, we describe the design of a novel primer set that can simultaneously detect all *Madurella* species in a single reaction. To achieve this goal, we conducted a comparative genome analysis that enabled us to design and evaluate primer sets targeting candidate genes common to *Madurella* species.

A total of 37 fungal strains (including 22 species) and five bacterial strains (including five species) known to cause mycetoma [3, 4] were used in this study, as listed in Table 1. These strains have been preserved at the Medical Mycology Research Center (MMRC; Chiba University, Japan) as an IFM collection through the National Bio-Resource Project (Japan) or at the Mycetoma Research Center (MRC) (Khartoum University, Khartoum, Sudan). Otherwise, other strains were obtained from the culture collections of the Westerdijk Fungal Biodiversity Institute (CBS; Netherlands), the Biological Resource Center, National Institute of Technology and Evaluation (NBRC; Japan), the American Type Culture Collection (ATCC; United States), the Fungal Genetics Stock Center (FGSC; United States), and the Japan Collection of Microorganisms, RIKEN BioResource Research Center (JCM; Japan).

**Table 1:** Fungal and bacterial strains used in this study

Species	Strain No
Fungi	
*Madurella mycetomatis*	IFM 46458
	IFM 68169 (= MRC No. 6)
	IFM 68172 (= MRC No. 14)
	IFM 68173 (= MRC No. 16)
	IFM 68174 (= MRC No. 21)
	IFM 68175 (= MRC No. 22)
	IFM 68176 (= MRC No. 26)
	IFM 68177 (= MRC No. 31)
	IFM 68178 (= MRC No. 33)
	IFM 68239 (= MRC No. 10)
	IFM 68240 (= MRC No. 16)
	IFM 68243 (= MRC No. 28)
	IFM 68244 (= MRC No. 32)
*Madurella pseudomycetomatis*	IFM 46460
*Madurella tropicana*	CBS 201.38^T^
*Madurella fahalii*	CBS 129176^T^
	IFM 68170 (= MRC No. 9)
	IFM 68171 (= MRC No. 13)
	IFM 68242 (= MRC No. 25)
*Aspergillus fumigatus*	Af293 (= FGSC A1100)
*Aspergillus niger*	IFM 68248
*Aspergillus nidulans*	IFM 68245 (= MRC No. 4)
*Aspergillus terreus*	IFM 67509
*Chaetomium globosum*	CBS 148.51^T^
*Chaetomium rectangulare*	CBS 126778^T^
*Curvularia lunata*	IFM 64654
*Exophiala jeanselmei*	IFM 67393
*Falciformispora senegalensis*	CBS 196.79^ T^
*Falciformispora tompkinsii*	CBS 200.79
*Fusarium oxysporum*	IFM 61457
*Fusarium solani*	IFM 67677
*Neocosmospora falciformis*	CBS 475.67
*Neotestudina rosatii*	CBS 331.78
*Sarocladium kiliense*	CBS 158.61
*Scedosporium apiospermum*	IFM 66388
*Thermothielavioides terrestris*	CBS 355.66 (= NBRC 9121)
*Trichophyton rubrum*	IFM 66913
Bacteria	
*Actinomadura madurae*	IFM 0585^ T^ (= JCM 7436^ T^)
*Actinomadura pelletieri*	IFM 0590^ T^ (= JCM 3388^ T^)
*Nocardia asteroides*	IFM 10791
*Nocardia brasiliensis*	IFM 12361
*Streptomyces somaliensis*	IFM 11185

The bacterial and fungal genomic DNA samples were extracted using the benzyl chloride method, as described previously [9]. In addition, the genomic DNA samples of *M. mycetomatis* IFM 46458 and *M. pseudomycetomatis* IFM 46460 were further purified using ISOSPIN Plant DNA (Nippon Gene, Toyama, Japan) in accordance with the instructions of the manufacturer for the evaluation of LAMP primer sets. Additionally, the extracted genomic DNA samples of *M. tropicana* CBS 201.38^ T^ and *M. fahalii* CBS 129176^ T^ were obtained from the CBS. The DNA concentration of these DNA samples was determined by QuantiFluor ONE dsDNA System (Promega, Madison, WI, USA).

For genomic sequencing, *M. pseudomycetomatis* IFM 46460 was cultivated in Sabouraud medium, and its genomic DNA was extracted by the phenol–chloroform method and purified with a Genomic-tip 100/G column (Qiagen, Hilden, Germany) as described previously [10]. The extracted genomic DNA was sequenced by Genome-Lead Co., Ltd. (Kagawa, Japan) via the Illumina NovaSeq 6000 and Nanopore PromethION platforms (Oxford Nanopore Technologies [ONT], Cambridge, UK). The genome assembly and gene annotation were performed in accordance with the methods described previously, with some modifications [10]. Briefly, genome assembly was executed using the ONT long reads not mapped to the mitochondrial genome by NECAT software v.0.0.1 [11] and Flye software v.2.9.2 [12] with a genome size of 35 Mb; however, the genome coverage value of input reads used for Flye assembly was set to a coverage × 50 in comparison with the previous study. Finally, genome annotation was performed by using the Funannotate software v.1.8.15 pipeline (https://github.com/nextgenusfs/funannotate).

The genomic sequences of *Madurella* spp. and their closely related species, members of *Chaetomiaceae* and *Podosporaceae* [13, 14], were obtained from the DDBJ/EMBL/GenBank database through NCBI (https://www.ncbi.nlm.nih.gov/) and the JGI genome portal (https://genome.jgi.doe.gov/portal/). The details of the genomic sequences obtained from DDBJ/EMBL/GenBank are as follows: *M. mycetomatis* mm55 (accession: GCA_001275765.2) [15], *Madurella fahalii* IFM 68171 (accession: GCA_045866475.1) [10], *Chaetomium globosum* CBS 148.51 (accession: GCA_000143365.1) [16], *Podospora anserina* S mat + (accession: GCA_000226545.1) [17] and *Mycothermus thermophilus* UFV (accession: GCA_011316235.1) [18]. Additionally, the genomic sequences obtained from JGI were as follows: *Thermothielavioides terrestris* NRRL 8126 (ID: 1184802) [19] and *Canariomyces arenarius* (ID: 1018941) [20]. The orthologous genes were identified using annotated protein sequences through SwiftOrtho [21], with a minimum alignment coverage of 80% and a sequence identity cutoff of 50%. Subsequently, genes conserved exclusively among *Madurella* species were extracted from the SwiftOrtho results and further refined through an all-vs-all nucleotide-level comparison using VSEARCH [22], with a minimum sequence length of 500 bp and a nucleotide identity threshold of 90%. We used PrimerExplorer (https://primerexplorer.eiken.co.jp/e/) to design LAMP primer sets based on the genomic sequence of *M. mycetomatis.*

The LAMP reaction was performed in a 25 μL reaction volume containing template DNA, 40 pmol FIP and BIP, 20 pmol loop primers (LF and LB), and 5 pmol F3 and B3 in Loopamp DNA Amplification Reagent D (Eiken Chemical Co., Ltd; Japan). However, the reaction mixture of Primer #3 contained 20 pmol of three loop primers in comparison with the other primer sets (#1 and #2). Unless otherwise noted, the reaction was conducted at 66 °C for 60 min, and amplification was monitored in real-time by the change in turbidity in the LAMP reactions using LoopampEXIA (Eiken Chemical Co., Ltd; Japan). To determine the detection limit, genomic DNA solutions of *M. mycetomatis* IFM 46458 ranging from 100 pg to 100 fg were tested. In addition, 1 pg of the genomic DNAs of *M. pseudomycetomatis* IFM 46460, *M. tropicana* CBS 201.38 and *M. fahalii* CBS 129176 were used to assess both the sensitivity and specificity for *Madurella* species. Finally, 1 ng of genomic DNA was used per reaction tube to determine the optimal reaction temperature and to check specificity.

The chromosomal genome assembly of *M. pseudomycetomatis* IFM 46460 resulted in six scaffolds with a total length of 40,045,822 bp and an N50 value of 25,195,283 bp. Gene annotation identified 11,334 protein-coding genes, 180 tRNAs, and 79 rRNAs with a 99.6% BUSCO v.5.5.0 [23] score for eukaryote databases. A comparative genome analysis was conducted by using species evolutionarily close to the *Madurella* genus to identify genes common with and specific to the *Madurella* genus. A two-step screening protocol identified forty genes that were well-conserved across the genomes of *Madurella* genus members, as shown in Supplementary Table 1. Among these candidate genes, the LAMP primers we designed were specific for three genes (locus tag in *M. mycetomatis* mm55 [15]: MMYC01_204841, MMYC01_205181, MMYC01_210211), targeting gene regions with few or no mutations, as shown in Table 2.

**Table 2:** LAMP Primers used in this study

Region	Sequence (5′ to 3′)
Primer #1 (MMYC01_204841)	
F3	AGCGGACTTGGCCTCATC
B3	GTAATCGTGGCTGCATTTCCA
FIP (F1c/F2)	CTCTCGTTGGCTCGGCAGAG-CGCCCCTCAAAGCTGGAT
BIP (B1c/B2)	ACCGCCGATGCCTCGAATCCTA-TGTAGTTGTGGCGAATCTTTCC
LF	AGCTGCTCGGGCGGA
LB	TCTCGGGCACCGGGTG
Primer #2 (MMYC01_205181)	
F3	AGGGGCTCCCAGGAAATC
B3	TCGTCGCTGGAATCCTTCA
FIP (F1c/F2)	CAGTAGGCACAGTCCTCGATCCT-TCCATCAGCATCGCTAAACG
BIP (B1c/B2)	TTGGCATCAACATGCCCATGC-GATCTCCTCCTGGAGTCGG
LF	TCTTTGCCGCCCACGACATG
LB	TGTATGGCGAGAACGAAAAGGC
Primer #3 (MMYC01_210211)	
F3	ATCGTCCGTCGTCCGC
B3	CGTAATCCTCAGCTCCCA
FIP (F1c/F2)	AGTTGCGCTGCAGCCTGAC-GACCGAGCAAGGTGACAAAG
BIP (B1c/B2)	CACTCGCTTGTCGAGGAACTCA-TCCTAACGTCGTCGAAAACA
LF	GGGCGACCGCGTGTC
LB1	ACGACGCTGCTGCCG
LB2	AGAACGACGCCACCGC

**Fig. 1:**
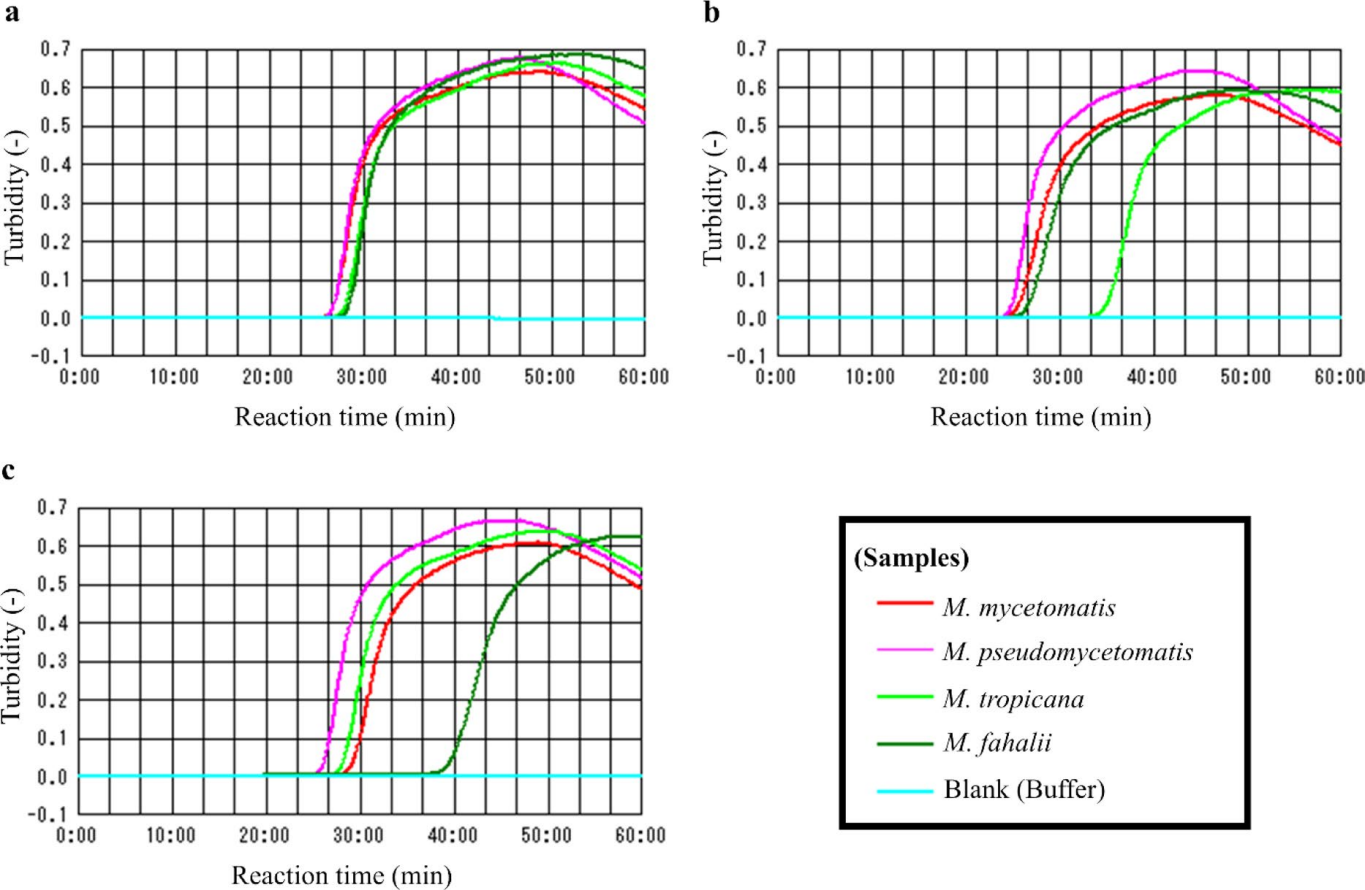
The specificity of designed LAMP primers, **a** #1 (for MMYC01_204841), **b** #2 (for MMYC01_205181) and **c** #3 (for MMYC01_210211). 1 pg of the extracted genomic DNAs derived from *Madurella* spp. were used as templates. The red, magenta, yellow-green, and green plots represent the data for *M. mycetomatis* IFM 46458, *M. pseudomycetomatis* IFM 46460, *M. tropicana* CBS 201.38^ T^, and *M. fahalii* CBS 129176^ T^, respectively. The light blue plot indicates a blank reaction mixture, in which Tris–HCl buffer was used as a template instead of DNA

The primer sets were tested by employing 100 pg of genomic DNA samples derived from *M. mycetomatis* IFM 46458 and *M. fahalii* CBS 129176 and running the LAMP reactions at temperatures ranging from 64 to 67 °C. The optimal temperature of the three primer sets was determined to be 66 °C, although they exhibited efficient amplification curves in all conditions, as shown in Supplementary Fig. 1. We determined the sensitivity of the three primer sets by employing 100 fg—100 pg of genomic DNA samples derived from *M. mycetomatis* IFM 46458. All three primer sets could detect up to 1 pg of genomic DNA, suggesting that they possess equal sensitivity, as shown in Supplementary Fig. 2. Subsequently, we evaluated the detection limits of other *Madurella* species. Although Primer #3 showed a reduced reactivity toward *M. fahalii*, these primer sets could also detect 1 pg of genomic DNA samples derived from three *Madurella* species (*M. pseudomycetomatis*, *M. tropicana*, *M. fahalii*) in approximately 45 min, as shown in Fig. 1.

Since the three LAMP primer sets showed equal sensitivity towards *Madurella*, we conducted further tests to assess their specificity towards other pathogens and select the optimal primer set. We conducted LAMP reactions using 1 ng of genomic DNA from fungal and bacterial species known as the mycetoma causative agents, as listed in Table 1 [3, 9]. Primers #1, #2 and #3 showed no non-specific (unexpected) reaction toward pathogenic microorganisms other than the *Madurella* species*.* However, Primer #2 failed to amplify the genomic DNA of the *M. fahalii strain* (IFM 68242), while the other two primer sets successfully detected all tested strains belonging to the *Madurella* species.

We successfully developed three primer sets which could detect genomic DNA derived from four *Madurella* strains belonging to four species. However, Primer #2 failed to amplify one *M. fahalii* strain. As for the reactivity of the LAMP reactions at lower genomic DNA concentrations, the amplification rates of Primer #1 for four *Madurella* species were comparable, while Primer #3 exhibited lower reactivity for low-concentration genomic DNA samples of *M. fahalii*. These results suggest that Primer #1 is the first choice for its practical application to clinical diagnosis. Additionally, the detection limit of this primer set is comparable to that of LAMP primers designed in a previous study [9]. However, since our study only tested the extracted DNA from culture isolate, the LAMP reaction using a grain should be performed to evaluate the practical use in diagnosis in the future. Moreover, to facilitate diagnosis in rural settings, it is also necessary to adapt or extend simple detection methods that do not require expensive instruments, such as colorimetric and fluorescent assays.

In conclusion, as described above, a new primer set can detect all known *Madurella* species in a single reaction tube, whereas previous studies required two or three separate reactions. This advantage should enable medical workers to avoid complex operation protocols and to reduce the cost per experiment. Thus, this primer set can replace the previous version as a molecular diagnostic kit.

## Supplementary Information

Below is the link to the electronic supplementary material.

Supplementary file1 (DOCX 1999 KB)

## Data Availability

The whole and mitochondrial genome sequences of *Madurella pseudomycetomatis* IFM 46460 were deposited at DDBJ/EMBL/GenBank under the accession numbers BAAIAC010000001-BAAIAC010000006 and AP043604, respectively. The raw sequencing reads were submitted to the SRA under the accession number DRA021621.
