# Supplementary material for: Simultaneous Detection of Four Madurella Species Using Loop-Mediated Isothermal Amplification (LAMP) for Eumycetoma Diagnosis
Source: Mycopathologia. 2025 Oct 29;190(6):105. doi: 10.1007/s11046-025-01019-4 (PMC12568910; doi:10.1007/s11046-025-01019-4)
Supplement: Supplementary file 1 — Supplementary file1 (DOCX 1999 KB) [file 11046_2025_1019_MOESM1_ESM.docx]

**Supplementary Material – Mycopathologia**

**Simultaneous detection of four *Madurella* species with a single primer set of** **loop-mediated isothermal amplification (LAMP) for eumycetoma diagnosis**

Isato Yoshioka, Ahmed Hassan Fahal, Doudou Sow, Satoshi Kaneko, Yugo Mori, Takashi Yaguchi^*^

| Supplementary Table 1 Genes common and specific to *Madurella* species identified by comparative genome analysis | | | |
| --- | --- | --- | --- |
| Locus tag in *Madurella* genome ^1)^ | | | Annotation ^2)^ |
| *M. mycetomatis* | *M. pseudomycetomatis* | *M. fahalii* |  |
| MMYC01_200287 | MPIFM46460_07989 | MFIFM68171_07637 | O-methyltransferase 12 |
| MMYC01_200288 | MPIFM46460_07990 | MFIFM68171_07636 | putative HC-toxin efflux carrier TOXA |
| MMYC01_200680 | MPIFM46460_03898 | MFIFM68171_04497 | protein BROTHER of FT and TFL 1 |
| MMYC01_200849 | MPIFM46460_02782 | MFIFM68171_07251 | putative cutinase 1 |
| MMYC01_201795 | MPIFM46460_06914 | MFIFM68171_00255 | protein Daple |
| MMYC01_202044 | MPIFM46460_04543 | MFIFM68171_03870 | ketosamine-3-kinase |
| MMYC01_202333 | MPIFM46460_06093 | MFIFM68171_01077 | NADPH dehydrogenase |
| MMYC01_202397 | MPIFM46460_04774 | MFIFM68171_06653 | ubiquinone/menaquinone biosynthesis C-methyltransferase |
| MMYC01_202472 | MPIFM46460_03223 | MFIFM68171_05213 | cytokinesis protein 3 |
| MMYC01_202654 | MPIFM46460_01547 | MFIFM68171_07255 | hypothetical protein |
| MMYC01_202654 | MPIFM46460_01547 | MFIFM68171_07255 | hypothetical protein |
| MMYC01_202920 | MPIFM46460_04877 | MFIFM68171_03506 | hypothetical protein |
| MMYC01_203253 | MPIFM46460_04214 | MFIFM68171_04190 | nitrile-specifier protein 2 |
| MMYC01_203329 | MPIFM46460_09557 | MFIFM68171_11059 | transcriptional activator Myb |
| MMYC01_203408 | MPIFM46460_07018 | MFIFM68171_00147 | hypothetical protein |
| MMYC01_204195 | MPIFM46460_06020 | MFIFM68171_01149 | DNA-(apurinic or apyrimidinic site) lyase 1 |
| MMYC01_204207 | MPIFM46460_03280 | MFIFM68171_05156 | 3-hydroxyisobutyryl-CoA hydrolase |
| MMYC01_204336 | MPIFM46460_06869 | MFIFM68171_00300 | delta(24)-sterol reductase |
| MMYC01_204708 | MPIFM46460_07347 | MFIFM68171_06269 | 3-hydroxybenzoate 6-hydroxylase |
| MMYC01_204841 | MPIFM46460_10584 | MFIFM68171_02134 | hypothetical protein |
| MMYC01_204953 | MPIFM46460_08145 | MFIFM68171_07475 | endoplasmic reticulum mannosyl-oligosaccharide 1,2-alpha-mannosidase |
| MMYC01_205045 | MPIFM46460_06622 | MFIFM68171_00685 | eukaryotic translation initiation factor 3 subunit M |
| MMYC01_205181 | MPIFM46460_07474 | MFIFM68171_05778 | vegetative incompatibility protein HET-E-1 |
| MMYC01_205578 | MPIFM46460_10651 | MFIFM68171_02649 | transcriptional activator hac1 |
| MMYC01_206031 | MPIFM46460_01313 | MFIFM68171_08924 | phosphomannomutase |
| MMYC01_206365 | MPIFM46460_02571 | MFIFM68171_06668 | hypothetical protein |
| MMYC01_207155 | MPIFM46460_07313 | MFIFM68171_08249 | flavohemoprotein |
| MMYC01_208756 | MPIFM46460_06020 | MFIFM68171_01149 | DNA-(apurinic or apyrimidinic site) lyase 1 |
| MMYC01_209044 | MPIFM46460_04774 | MFIFM68171_06653 | Ubiquinone/menaquinone biosynthesis C-methyltransferase UbiE |
| MMYC01_209176 | MPIFM46460_08834 | MFIFM68171_10361 | protein JSN1 |
| MMYC01_209254 | MPIFM46460_08200 | MFIFM68171_07425 | anucleate primary sterigmata protein B |
| MMYC01_209309 | MPIFM46460_03223 | MFIFM68171_05213 | cytokinesis protein 3 |
| MMYC01_209407 | MPIFM46460_10184 | MFIFM68171_01957 | Rho1 guanine nucleotide exchange factor 1 |
| MMYC01_209680 | MPIFM46460_05425 | MFIFM68171_08139 | erythronolide synthase, modules 1 and 2 |
| MMYC01_209681 | MPIFM46460_05424 | MFIFM68171_08140 | Isoflavone reductase P3 |
| MMYC01_209682 | MPIFM46460_05423 | MFIFM68171_08141 | hypothetical protein |
| MMYC01_209683 | MPIFM46460_05422 | MFIFM68171_08142 | verruculogen synthase |
| MMYC01_209924 | MPIFM46460_01277 | MFIFM68171_08963 | transmembrane 9 superfamily member 4 |
| MMYC01_210211 | MPIFM46460_08826 | MFIFM68171_10353 | hypothetical protein |
| MMYC01_210701 | MPIFM46460_08850 | MFIFM68171_10377 | forkhead protein sep1 |
| 1) Locus tags are based on the DDBJ/EMBL/GenBank database for the following strains: *M. mycetomatis* mm55 (accession: GCA_001275765.2), *M. pseudomycetomatis* IFM 46460 (accession: XXX.1), and M. *fahalii* IFM 68171 (accession: GCA_045866475.1). | | | |
| 2) Gene annotation was derived from the genomic sequence of *M. mycetomatis* mm55. | | | |


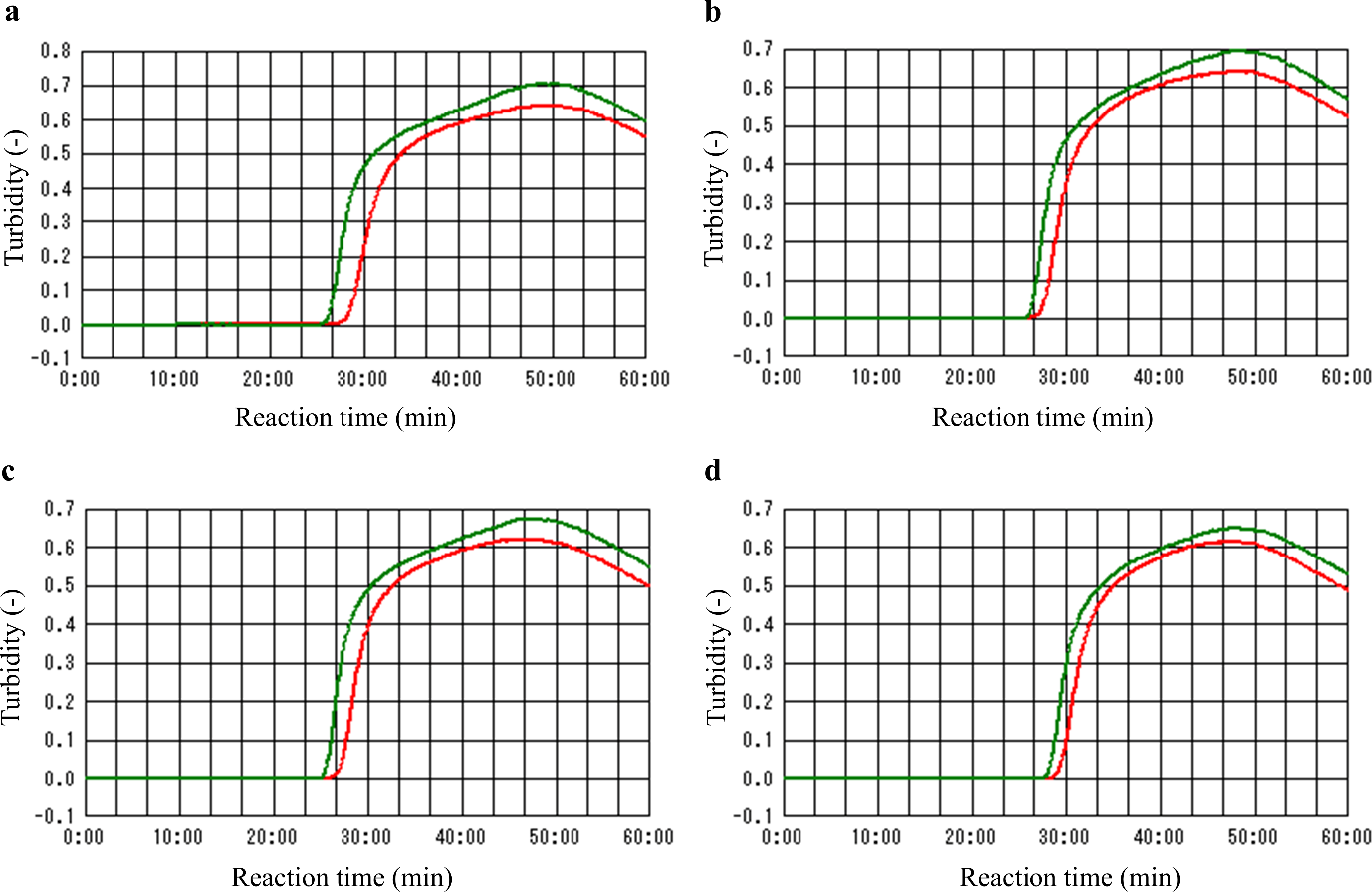

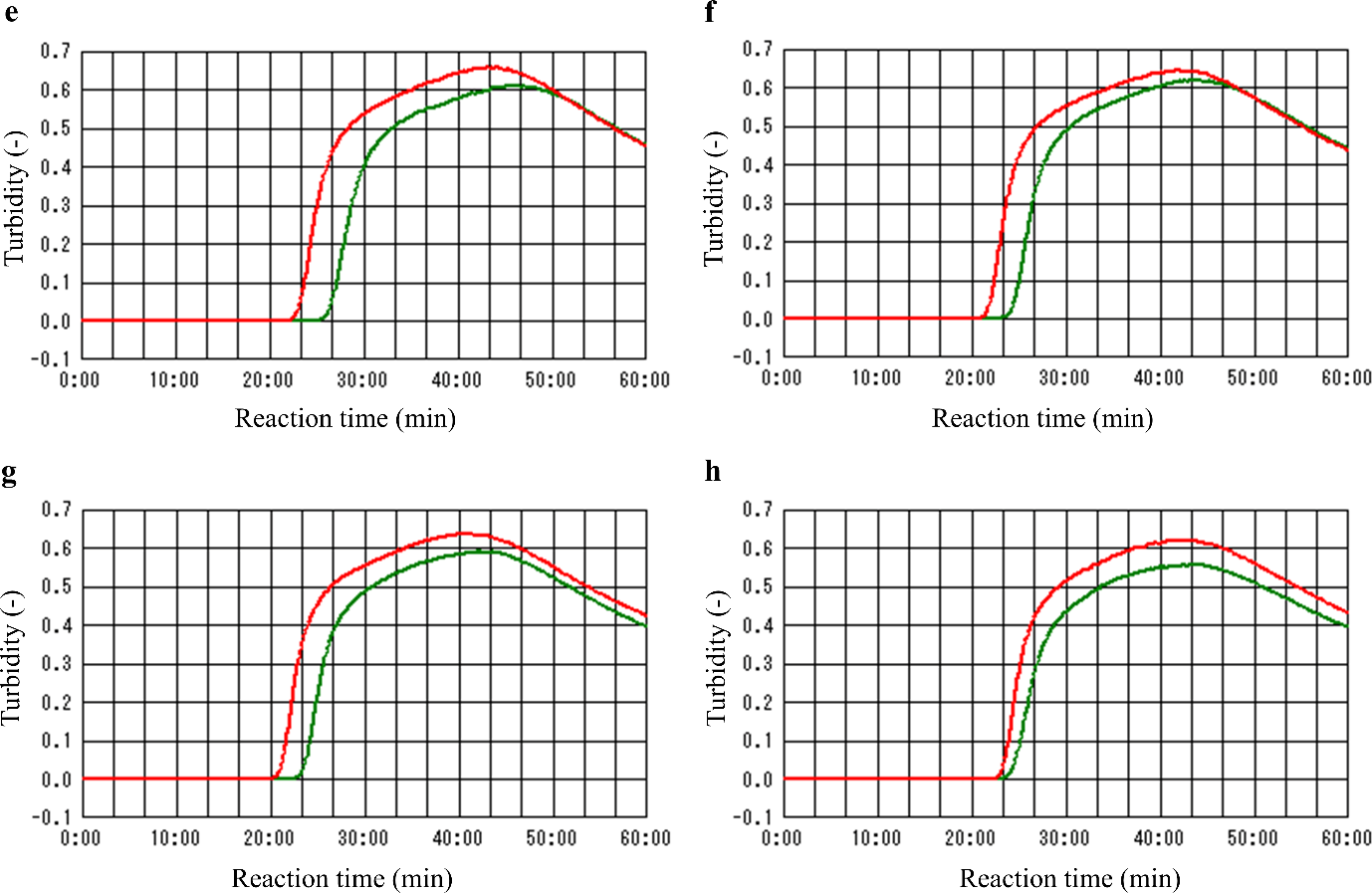


(Supplementary Figure 1; continued on next page)


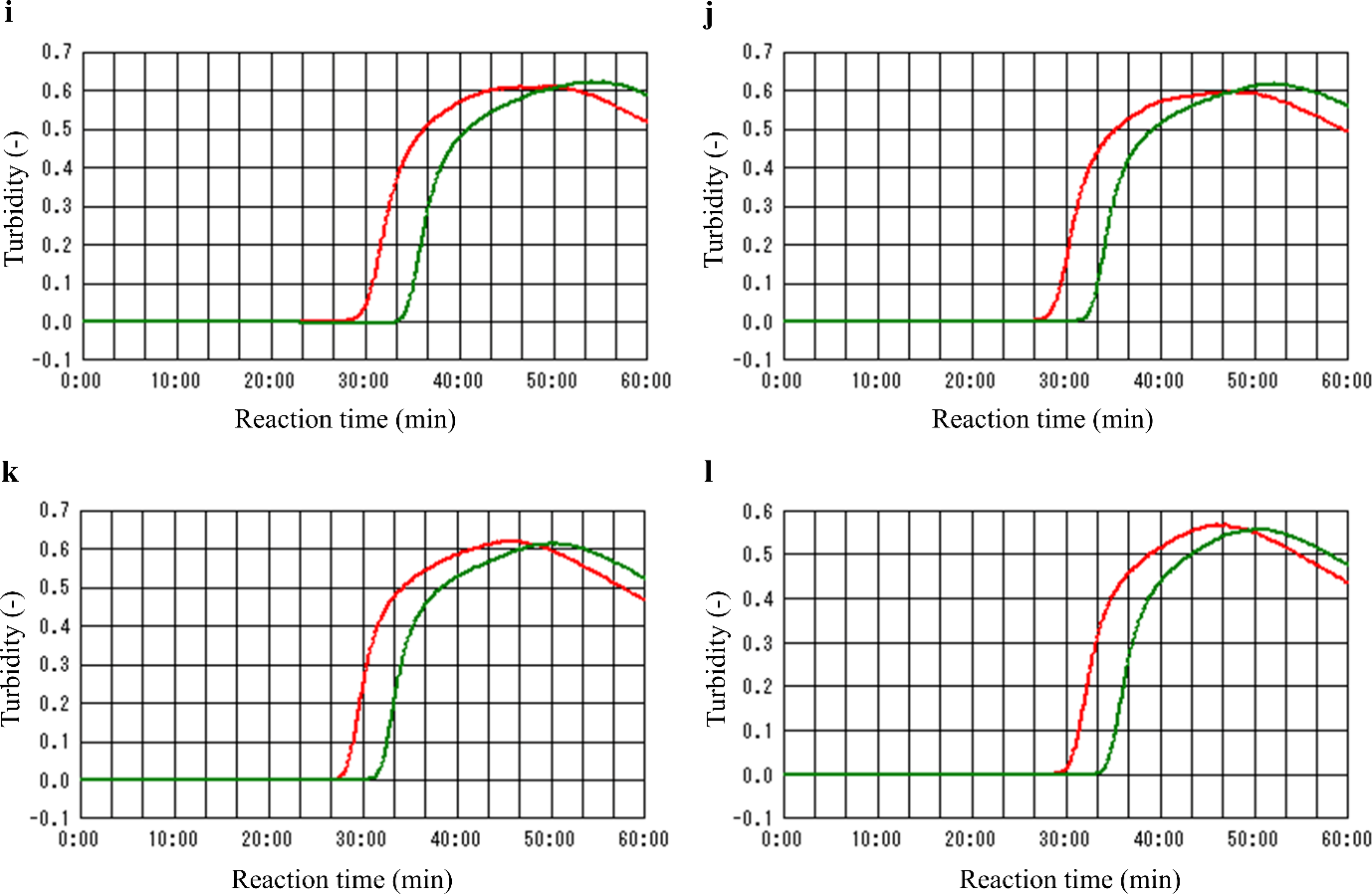


**Supplementary Figure 1**. Optimization of LAMP reaction temperature using primer #1 (a-d), #2 (e-h) and #3 (i-l). 100 pg of genomic DNAs of *Madurella myceromatis* IFM 46458 and *M. fahalii* IFM 68171 were used as a template. LAMP reaction was performed for 60 min at the reaction temperature at 64 °C (a,e,i), 65 °C (b,f,j), 66 °C (c,g,k) and 67 °C (d,h,l). The red line and green line represent the data of *M. nycetomatis* and *M. fahalii*, respectively.


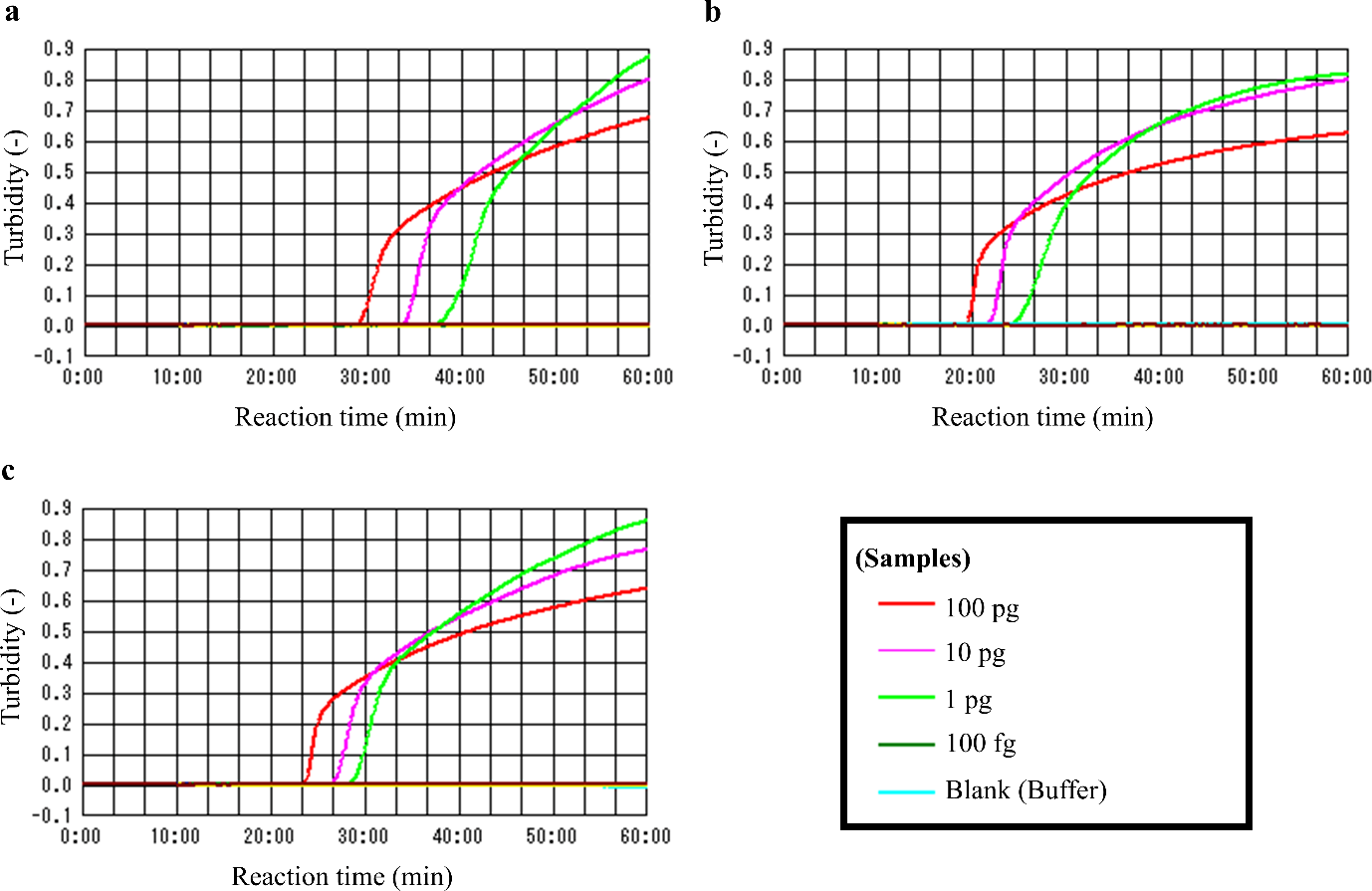


**Supplementary Figure 2**. The detection limits of designed LAMP primers, (a) #1, (B) #2 and (C) #3. The genomic DNAs of *M. mycetomatis* IFM 46458 were serially diluted by 10-fold, and 100 pg - 100 fg of DNAs were used as a template. LAMP reaction was performed at 66 °C for 60 min. The sample legends of the plots were represented on the bottom right panel, and a blank sample was prepared by adding Tris-HCl buffer instead of genomic DNA solution.
